# Cytomegaloviruses Exploit Recycling Rab Proteins in the Sequential Establishment of the Assembly Compartment

**DOI:** 10.3389/fcell.2018.00165

**Published:** 2018-12-04

**Authors:** Pero Lučin, Ljerka Kareluša, Gordana Blagojević Zagorac, Hana Mahmutefendić Lučin, Valentino Pavišić, Natalia Jug Vučko, Silvija Lukanović Jurić, Marina Marcelić, Berislav Lisnić, Stipan Jonjić

**Affiliations:** ^1^Department of Physiology and Immunology, Faculty of Medicine, University of Rijeka, Rijeka, Croatia; ^2^University North – University Center Varaždin, Varaždin, Croatia; ^3^Department of Histology and Embryology, Faculty of Medicine, University of Rijeka, Rijeka, Croatia

**Keywords:** cytomegalovirus, virion assembly compartment, endosomal recycling compartment, Rab proteins, Rab cascades

## Abstract

Cytomegaloviruses (CMV) reorganize membranous system of the cell in order to develop a virion assembly compartment (VAC). The development starts in the early (E) phase of infection with the reorganization of the endosomal system and the Golgi and proceeds to the late phase until newly formed virions are assembled and released. The events in the E phase involve reorganization of the endosomal recycling compartment (ERC) in a series of cellular alterations that are mostly unknown. In this minireview, we discuss the effect of murine CMV infection on Rab proteins, master regulators of membrane trafficking pathways, which in the cascades with their GEFs and GAPs organize the flow of membranes through the ERC. Immunofluorescence analyzes of murine CMV infected cells suggest perturbations of Rab cascades that operate at the ERC. Analysis of cellular transcriptome in the course of both murine and human CMV infection demonstrates the alteration in expression of cellular genes whose products are known to build Rab cascades. These alterations, however, cannot explain perturbations of the ERC. Cellular proteome data available for human CMV infected cells suggests the potential role of RabGAP downregulation at the end of the E phase. However, the very early onset of the ERC alterations in the course of MCMV infection indicates that CMVs exploit Rab cascades to reorganize the ERC, which represents the earliest step in the sequential establishment of the cVAC.

## Introduction

**FIGURE 1:**
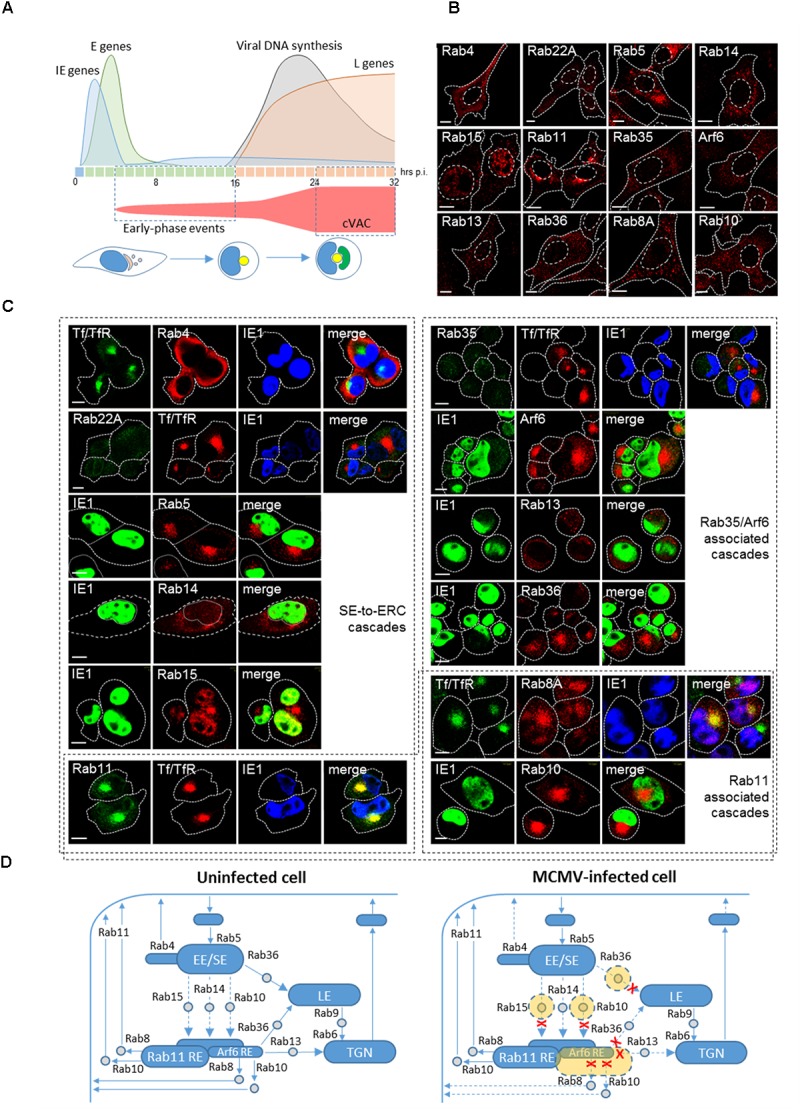
Reorganization of the ERC in the early phase of CMV infection. **(A)** *Kinetics of MCMV gene expression and development of cytoplasmic virion assembly compartment (cVAC)*. Expression of MCMV genes is organized in the immediate early (IE, blue, first hour), early (E, green, 2–16 hpi) and late (L, orange, after initiation of viral DNA replication) phase and is associated with perturbation of cellular functions throughout the entire replication cycle. The kinetics and the volume of membranous organelle reorganization that lead to the development of the cVAC are outlined in red. E-phase events lead to reorganization of the endosomal recycling compartment (ERC) and the Golgi, which at the end of E-phase (16 hpi) forms a compact juxtanuclear structure that represents the core of the cVAC (yellow). Viral DNA synthesis and expression of L-genes lead to the cytoplasmic accumulation of viral tegument proteins and vacuolar accumulation of viral glycoprotein as a cap (green) that surrounds the core at 24 hpi and later. *Confocal immunofluorescent images of membrane-bound small GTPases that that control membrane trafficking through the ERC in uninfected cells* **(B)** *and MCMV-infected cells* **(C)** *at the end of the E phase of infection (16 hpi)*. Antibody reagents and experimental procedures were described in the article by Karleuša et al. (2018). Tf/TfR represent transferrin receptor (TfR) after 60 min internalization (15–16 hpi) of AF^488^-labeled transferrin (Tf). IE1 represents immediate-early 1 protein of MCMV that is expressed in the nucleus of the infected cell. Fine dotted and dashed lines indicate cell and nuclear borders, respectively. Bars, 10 μm. **(D)** *Schematic presentation of incoming and outgoing trafficking pathways at the ERC of uninfected and MCMV-infected cells*. Structures that form stable compartments visible by the conventional confocal microscopy are presented in blue and subvisible endosomal intermediates in gray. Structures expanded by MCMV infection are presented in orange and pathways that may be inhibited by dashed lines and red crosses. EE/SE, early/sorting endosome. RE, recycling endosome; TGN, the trans-Golgi network; LE, late endosomes.

Cytomegaloviruses, like other herpesviruses, induce extensive reorganization of cellular functions, including the rearrangement of the membranous system (Johnson and Baines, 2011; Henaff et al., 2012). CMV replication program is executed through the sequential expression of viral genes organized into at least three phases: immediate early (IE), early (E) and late (L) phase. In human CMV (HCMV) infected fibroblasts, IE events are executed at 6–24 h post infection (hpi), E events at 12–48 hpi, and L events at 48–96 hpi, followed by assembly and release of infectious virions at 72–96 hpi (Gurczynski et al., 2014). In fibroblasts infected with murine CMV (MCMV), a well-established model for studying CMV infection *in vivo* and *in vitro*, the whole cycle is much shorter, and the first viral progeny is produced 24–30 hpi (Shellam et al., 2006). The IE phase is restricted to 1–2 hpi and E phase to 2–16 hpi (Figure 1A). At 16 hpi viral DNA synthesis is initiated, and a large number of late genes is transcribed (Marcinowski et al., 2012). Transcriptome analysis of both HCMV- (Tirosh et al., 2015) and MCMV- (Marcinowski et al., 2012; Lisnic et al., 2013) infected cells and proteome analysis of HCMV-infected cells (Weekes et al., 2014; Tirosh et al., 2015; Jean Beltran et al., 2016) demonstrated that infection is associated with alterations of numerous cellular gene expression.

One of the goals of CMV-induced cellular perturbations is the establishment of a cytoplasmic environment for assembly of newly formed virions. After synthesis of all components in the L phase, nascent capsids are assembled in the nucleus, released into cytosol through a process of envelopment with the nuclear membrane (primary envelopment), followed by a series of cytoplasmic steps that involve tegumentation, envelopment at modified membraneous organelles (secondary envelopment) and virion egress (rev. by Tandon and Mocarski, 2012). The sequence of cytoplasmic events and the secondary envelopment occur at the pre-formed aggregate of membranous structures known as cVAC.

The development of cVAC is initiated immediately upon infection. It involves reorganization of the Golgi and the endosomal system in a proper sequence of required membranous structures around the cell center (Tandon and Mocarski, 2012). The reorganization can be divided into at least two stages. In MCMV infected cells, the first stage advances throughout the E phase of infection (3–16 hpi) and the second stage takes place in the L phase (16–24 hpi) until the first progeny virions are released (Figure 1A). Studies of MCMV (Ilić Tomaš, 2010; Karleuša et al., 2018) and HCMV infection (Krzyzaniak et al., 2009; Close et al., 2018; Zeltzer et al., 2018) point that one significant target of membranous perturbation is the endosomal recycling system and the ERC. The ERC is also exploited by other viruses for various aspects of viral pathogenesis (Vale-Costa and Amorim, 2016; Cruz and Buchkovich, 2017).

The ERC represents one branch in the endosomal maturation and highly dynamic router of membrane flow that undergoes through a series of transitions regulated by the cascade recruitment of Rab proteins and their effectors (Grant and Donaldson, 2009). Thus, in this article, we focus on the alteration of Rab protein cascades that regulate the ERC in the E phase of CMV infection and the early stage of cVAC development. We do not, however, address other important perturbations that coincide with it (i.e., perturbation of TGN). We believe that analysis of Rab cascades in CMV infected cells not only contribute to the understanding of the development of cVAC but also may elucidate the physiological interactions of membrane shapers under physiological expression levels.

## CMV Assembly Compartment is Initiated in the Early Phase of Infection

A study in MCMV infected cells demonstrated that the endosomal rearrangement and dislocation of the Golgi are initiated already at 3–5 hpi (Karleuša et al., 2018). Analysis of endosomal markers expression indicates that the endosomal rearrangement involves perinuclear aggregation of membranous structures derived from EE/SEs, EE/SEs-to-ERC intermediates, and the ERC. The initial structure observed at 6 hpi is further expanded at 16 hpi and maintained at later stages (i.e., 30 hpi) when the cVAC is fully developed (not shown), suggesting that the E-phase events involve the development of the cVAC core (Figure 1A). The endosomal rearrangement involves alteration of endosomal routes at the stage of early/sorting endosomes (EE/SE), as is also recently observed in HCMV infected cells (Zeltzer et al., 2018). It causes retention and merging of both clathrin-dependent endocytic (CDE; i.e., Tf/TfR) and clathrin-independent endocytic (CIE; i.e., MHC-I) cargo molecule trafficking (Lučin et al., 2015; Karleuša et al., 2018; Zeltzer et al., 2018). Recently described model (Close et al., 2018) predicts that HCMV also changes the flux through the ERC pathway and thereby accumulates transport machinery into discrete regions distributed throughout the cVAC.

## Cmv Assembly Compartment Involves Recycling Endosomes

Multiple studies on the organization of the cVAC during HCMV infection suggested that CMV infection reorganize REs into a perinuclear cluster that form the core of the cVAC (Sanchez et al., 2000; Homman-Loudiyi et al., 2003; Das et al., 2007; Krzyzaniak et al., 2009; Cepeda et al., 2010; Das and Pellett, 2011; Hook et al., 2014). These studies demonstrated retention of the recycling cargo (TfR) in perinuclear cluster of Rab11- and Arf6-positive vesicles and tubules. Membranes stained with these markers of REs blended with vesicles or membranes bearing markers of EE/SEs (i.e., Rab5, EEA1, Hrs), which often highly colocalized with RE markers. The perinuclear cluster impacts upon the nucleus and develops its reniform shape (Tandon and Mocarski, 2012). Late endosomal markers are excluded from the core and surround the perinuclear cluster together with fragmented Golgi elements (Cepeda et al., 2010; Das and Pellett, 2011; Rebmann et al., 2016). Despite many studies, the biogenesis of the core remained unclear. A study demonstrating the recruitment of a Rab11 effector that regulates biogenesis of the ERC (Krzyzaniak et al., 2009) confirmed the central role of the ERC in the biogenesis of the cVAC core.

## The ERC is Composed of Heterogeneous Subsets of Recycling Endosomes Regulated by Rab Cascades

The ERC represents a complex of heterogeneous subsets and functionally linked populations of REs that include relatively large perinuclear structures, tubular REs and a number of small transport intermediates (Xie et al., 2016). Several studies suggest that the ERC is composed of regularly present Rab11-positive membrane subset and an expandable, more pericentriolar, Arf6-positive membrane subset (Kobayashi and Fukuda, 2013). It appears that the ERC stratification reflects both biochemical composition and functional segregation of membrane trafficking (Xie et al., 2016), including multiple sorting functions toward the recycling, retrograde and exocytic route (Uchida et al., 2011).

Rab GTPases are master regulators of membrane traffic that control distinct steps in membrane flow by recruiting diverse effector proteins (rev. by Wandinger-Ness and Zerial, 2014). Inactive Rab proteins are present in the cytosol in the GDP form and attach to membranes of the endosomal system when their activation components become available during membrane flow program. At membranes, Rab proteins are activated by GEFs and inactivated by attachment of guanine-nucleotide activating proteins (GAPs) that facilitate hydrolysis of GTP and thereby detach Rab proteins from the membrane. Rab activation and inactivation cycle at membranes is organized as a cascade of programmed series of recruitment of Rabs, GEFs, and GAPs. GEF proteins control the site of Rab recruitment and GAP proteins control the lifetime of active Rab at the membrane.

The membrane flow into the ERC involve a transition of Rab5-positive EE/SEs to Rab11-REs and Arf6/Rab8-REs (Homma and Fukuda, 2016) and the flow of membranes from the TGN (Progida and Bakke, 2016). Trafficking between EE/SEs and the ERC may be regulated by Rab10 (Liu and Grant, 2015), Rab14 (Linford et al., 2012), and Rab15 (Strick and Elferink, 2005). The Arf6/Rab35 regulatory axis of recycling from the ERC, in which they act antagonistically (Klinkert and Echard, 2016), may regulate the size of the Arf6-REs within the ERC. Rab35 cascade which involves transition toward downstream Rabs (Rab8A, Rab10, Rab13, Rab36) (Kobayashi et al., 2014) may regulate exit out of the Arf6-RE. Additionally, Rab8A and Rab10 may also regulate exit from Rab11-REs (Homma and Fukuda, 2016). The outgoing flow of membranes at the Rab-11-REs involves complex and sequential activation of Rab-to-Arf cascades, as recently described for Rab4-orchestrated cascades at SEs (D’Souza et al., 2014) and recruitment of EHD proteins (Zhang et al., 2012). The trafficking routes to, within, and out of the ERC and expected sites of action of Rab proteins are depicted in Figure 1D.

## CMV Exploits Rab Cascades to Reshape the ERC and Redirect the Membrane Flow

The sequences of regulatory networks that control membrane flow through the ERC in uninfected cells as well as the sequence of alterations that lead to the final establishment of the cVAC in CMV infected cells are far from being completely understood. A small piece of evidence indicates that alteration of Rab recruitment is exploited by CMVs as an integral part of a complex mechanism that leads to the development of the cVAC.

In uninfected fibroblasts, Rab proteins and their effectors are mainly cytosolic, and only those that decorate major endosomal organelles display distinguishable structures visible by conventional confocal microscopy (Figure 1B). Many transport intermediates are dimly fluorescent and difficult to distinguish, especially under conditions of physiological expression levels. For example, Rab10-positive vesicles that mediate transport between endosomes are hardly detectable in static images (Babbey et al., 2006). In MCMV infected cells, however, the rearranged membranous organelles positive for several Rab proteins, including Rab10, are concentrated as a perinuclear aggregate (Figure 1C). Enrichment of Rab5-positive membranes indicates the concentration of EE/SEs and the absence of Rab22A (Figure 1C) suggests dysregulation of Rab22A-to-Rab5 cascade known to control exit from SEs (Magadán et al., 2006; Zhu et al., 2009). Displacement of Rab4 toward the cell periphery (Figure 1C) indicates reduced recruitment of small GTPase cascade (Rab4-Arl1-Big1/2-Arf1/3 cascade) at EEs described by D’Souza et al. (2014) that controls recycling. Enrichment of Rab10- and Rab15-positive membranes (Figure 1C) indicates expansion of intermediates that mediate flow from EEs to the ERC and suggests alteration of downstream cascades, whereas enrichment Arf6-, and Rab8-positive membranes and the absence of Rab35 membranes (Figure 1C) indicates alteration of the outgoing flow of membranes from the ERC.

In addition to the conditions of facilitated exit from EE/SEs, it appears that CMV infection inhibits entry and expands intermediates that mediate membrane flow toward the ERC. Control of the trafficking between EE/SEs and REs has been assigned to Rab10 (Shi et al., 2010), Rab14 (Linford et al., 2012) and Rab15 (Strick and Elferink, 2005), through often subvisible intermediates in uninfected cells. The cascade transition of Rab5 to either Rab14 or Rab15 has not been demonstrated, whereas the cascade between Rab5 and Rab10 has been demonstrated recently. In *C. elegans* epithelial cells Rab5 recruits an effector which promote interaction of Rab10 GEF with Rab10 (Liu et al., 2018), whereas Rab10 recruits GAP for Rab5 at the endosomes (Liu and Grant, 2015) thereby facilitating the exit of recycling cargo from SEs. Thus, accumulation of Rab10-positive membranes in the perinuclear aggregate of MCMV-infected cells (Figure 1C) indicates expansion of Rab10-positive intermediates that mediate transport into the ERC and suggest altered recruitment of Rab10 GAP by a downstream cascade. Given that Rab10 negative feedback controls entry of CIE cargo into the ERC (Liu et al., 2018), the observed retention of CIE cargo (Lučin et al., 2015; Karleuša et al., 2018) may occur in these intermediates and could explain inhibitory effect of MCMV infection on their recycling. Similarly, expansion of Rab15-positive intermediates in the perinuclear aggregate (Figure 1C) may explain inhibitory effect of MCMV infection on recycling of CDE cargo (Karleuša et al., 2018), since it has been shown that Rab15 controls entry of TfR from SEs into the ERC (Strick and Elferink, 2005). Although, it has been shown that Rab14 controls trafficking and recycling of CDE cargo at intermediates between EE/SEs and the ERC (Linford et al., 2012), it appears that MCMV infection does not affect these intermediates and that Rab14 is recruited to more peripheral endosomal compartments (Figure 1C), which mediates transport between EEs and the TGN (Reed et al., 2013).

Accumulation of Arf6-positive compartments (Figure 1C) in the perinuclear aggregate suggests the overactivation of Arf6 and expansion of Arf6-REs within the ERC. Overactivation of Arf6 is associated with high recruitment of EPI64 (data not shown), which is a known GAP for Rab35, and inhibition of endosomal recycling (Klinkert and Echard, 2016). Arf6 activation at the ERC could be controlled by Rab35, Rab10, and Rab8. The well-established feedback loop between Arf6 and Rab35, in which Arf6 recruits a GAP for Rab35 and Rab35 recruits a GAP for Arf6, operates at the cell periphery and within the ERC (Klinkert and Echard, 2016). This loop seems to be altered in MCMV infected cells since Rab35 (Figure 1C) and its effector that shut off Arf6 (not shown) were not recruited at the perinuclear aggregate. Apparently, overactivation of Arf6 is essential for the progression of CMV infection. The extent of Arf6 activation may be constrained by both Rab10 and Rab8. In *C. elegans* cells both Rab10 and Rab8 recruit the same GAP as Rab35 (Shi and Grant, 2013) to control Arf6 activation. However, a recent study in neuron-like cells (Homma and Fukuda, 2016) suggest that Rab8 can be activated at Arf6 REs and Rab10 at Rab11-REs. Although in polarized trafficking Rab8 and Rab10 may act at different locations, it has been suggested that in non-polarized cells they could function redundantly (Shi et al., 2010). Thus, both Rab10 and Rab8 may constrain overactivation of Arf6 and expansion of Arf6-REs in MCMV infected cells. However, Rab8 is absent from the perinuclear aggregate at 6 hpi (Karleuša et al., 2018) and highly enriched at 16 hpi (Figure 1C), indicating the temporal sequence in Arf6 overactivation. Dysregulation of the Arf6 axis has been recently described in the early phase of HCMV infection (Zeltzer et al., 2018).

Overactivation of Arf6 at the ERC may turn off Rab35-associated downstream cascades, which operate at Arf6-REs but not at Rab11-REs (Kobayashi et al., 2014). Rab35 through its effector recruits Rab8A, Rab10, Rab13, and Rab36 at the ERC and thereby promotes exit from the Arf6-REs (Rahajeng et al., 2012; Kobayashi et al., 2014). However, only Rab13 was not recruited to the perinuclear aggregate (Figure 1C) indicating that Rab8A, Rab10, and Rab36 are recruited at other locations within the perinuclear aggregate. The downstream routes in the Rab35 cascade are not well characterized, however, it is known that Rab8A and Rab10 may promote recycling from REs by recruiting EHD proteins (Grant and Caplan, 2008; Allaire et al., 2010). Although both Rabs are recruited to the perinuclear aggregate, it seems that the recycling route at the ERC controlled by Rab35 does not function in MCMV infected cells. Similarly, the route based on the recruitment of Rab13 and Rab36 are shut off in MCMV infected cells. Both Rab13 and Rab36 may be involved in the control of trafficking between TGN and REs (Nokes et al., 2008), and Rab36 also in trafficking from EE/SEs and REs to LEs (Matsui et al., 2012; Nottingham et al., 2012). Thus expansion of Rab36-positive membranes in the perinuclear aggregate of MCMV-infected cells (Figure 1C) could represent intermediates that link EE/SEs with LEs.

Although enrichment of Rab11-REs at the perinuclear aggregate (Figure 1C) and inhibited recycling of CDE cargo (Karleuša et al., 2018) may suggest an alteration of outgoing flow, it cannot be explained by altered recruitment of downstream Rabs. The Rab11 function is associated with the docking of recycling intermediates to the plasma membrane (Takahashi et al., 2012), whereas exit from Rab11-REs involves multiple factors, including recruitment of Rab10 and Rab8 (Homma and Fukuda, 2016) and EHD proteins (Grant and Caplan, 2008). Enhanced recruitment of Rab10 and Rab8 at the perinuclear aggregate is not consistent with the alteration of outgoing membrane flow at Rab11-positive membrane domains since activation of these Rabs facilitates exit from Rab11-REs.

Altogether, analysis of the perinuclear aggregate in the E phase of MCMV infection indicates that CMV exploits multiple Rab proteins to reorganize the ERC into the core of the cVAC. Possible targets of MCMV infection are depicted in Figure 1D.

## Cmv Infection Affect Transcription and Expression Level of Rab Proteins and Rab-Gaps

**FIGURE 2:**
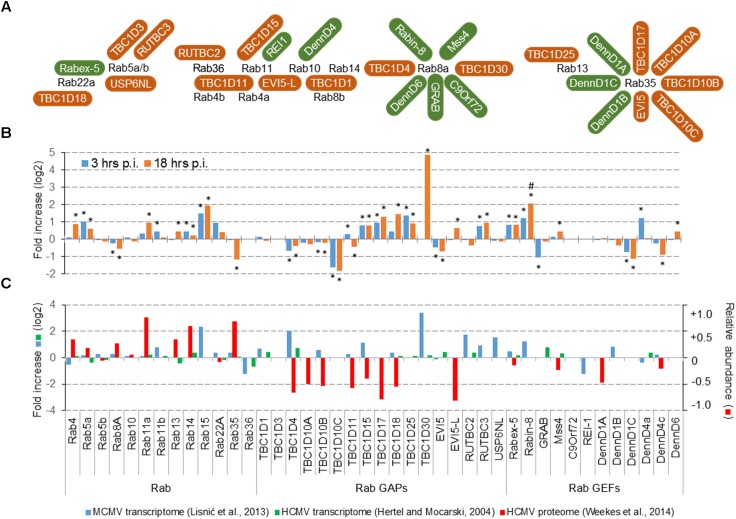
Effect of CMV infection on the expression of Rab proteins that control endosomal recycling route and their known GEFs and GAPs. **(A)** Rab proteins that control endosomal recycling, their known GAPs (brown) and GEFs (green). The illustration outlines the known interaction reviewed recently by Müller and Goody (2018). **(B)** Transcriptome of dendritic cells DC2.4 at 3 and 18 h post infection (p.i.). The data is represented as the fold change (log2) of gene expression in wild-type MCMV infected cells relative to the mock-infected cells. Shown are the data for all differentially expressed genes. A significant difference to mock-infected cells at *p*-value smaller than 0.1 (^∗^) and 0.05 (#) is shown above or below bars. Both, mock and MCMV-infected cells were analyzed in three replicates. **(C)** Comparative analysis of the transcriptome data in MCMV- (Lisnic et al., 2013) and HCMV- (Hertel and Mocarski, 2004) infected cells, and proteome data in HCMV-infected cells (Weekes et al., 2014). The transcriptome data for MCMV represent pooled samples from all phases of infection, the transcriptome data for HCMV represent samples at the end of the E-phase of infection (50 h p.i.), and the proteome data for HCMV represent samples at 40 h p.i. Left axis relates to the transcriptome data (log2 fold change), and the right axis relates to the proteome data (the percentage of change relative to mock-infected cells).

One approach used by CMVs could be manipulation with the amount of proteins that shape membranous organelles, either by up- and down-regulation of transcription, translation or protein degradation. Transcriptome analysis of MCMV infected cells at 3 and 18 hpi (Figure 2B) demonstrated alterations of expression of Rab, RabGEF, and RabGAP genes that control the recycling system (Figure 2A). Many of these alterations may be considered statistically significant (^∗^) when the cut-off value was adjusted to *p* < 0.1, whereas none of them was significant at the cut-off *p* < 0.05. Even upregulation of TBC1D30 observed at 18 hpi is insignificant at *p* < 0.05, because of the very low level of transcript in uninfected cells. However, these alterations, as well as most of the alterations observed in the previous study (Lisnic et al., 2013), correlated by little with alterations observed in HCMV-infected (Figure 2C).

Given that most of altered Rab protein genes are upregulated, downregulation of Rab gene expression is not a mechanism that could explain membrane reshaping in the E-phase of infection. On the contrary, upregulation of some genes encoding GEFs and GAPs may correlate with the low recruitment of Rab13, Rab22a, and Rab35 and could explain some of the alterations observed in the E phase of MCMV infection. Thus, manipulation with the expression level of regulatory proteins could be a potential target of CMVs. This conclusion may be supported by observations from temporal quantitative proteome analysis in HCMV infected cells at the end of E phase (Weekes et al., 2014), which demonstrated significant of several GAP proteins (Figure 2C). Given that proteome alterations do not correlate with the transcriptome alterations, it seems that CMVs can regulate the organization of the ERC by modulating degradation of Rab-cascades components, especially by enhancing degradation RabGAP proteins.

Altogether, immunofluorescence, proteome, and transcriptome analysis suggest that the main alteration of CMVs in the E phase of infection could be the recruitment of components of Rab cascades and that targeting of RabGAP proteins could be a mechanism exploited by CMVs in order to reshape membranous system of the cell.

## Concluding Remarks

Analysis of the perinuclear endosomal aggregate that is established at the end of the E phase of CMV infection indicates that CMVs exploit Rab cascades to take over the control at the ERC trafficking routes and thereby initiate the establishment of the cVAC. Although a plethora of data in the last decade provided clues about Rab cascades, many components remain unidentified and functional networks that construct the cascades poorly characterized *in vivo* under physiological conditions (Pfeffer, 2017). The analysis of the early events in the course of CMV infection display small fragments of this map and suggests that CMV infection could be a useful tool in analyzing Rab cascades under physiological levels of Rab protein expression.

## Author Contributions

PL conceived and coordinated the study, carried out image analysis, conceived figure presentation, and drafted the manuscript. HL and GZ coordinated the study, established immunofluorescence protocols, and carried out recycling analysis. LK, VP, NV, and SiJ carried out immunofluorescence and imaging studies. BL and StJ performed the transcriptome analysis. All authors read and approved the final manuscript.

## Conflict of Interest Statement

The authors declare that the research was conducted in the absence of any commercial or financial relationships that could be construed as a potential conflict of interest.

## Abbreviations

CMV: cytomegalovirus
cVAC: cytoplasmic virion assembly compartment
EE/SEs: early/sorting endosomes
ERC: endosomal recycling compartment
GAP: guanine-nucleotide activation protein
GEF: guanine-nucleotide exchange factor
HCMV: human cytomegalovirus
MCMV: murine cytomegalovirus
REs: recycling endosomes
Tf/TfR: transferrin bound to the transferrin receptor
TGN: the trans-Golgi network
